# Morphologic and functional features of left atrial appendage in Iranian population: an echocardiographic study

**DOI:** 10.15171/jcvtr.2019.38

**Published:** 2019-08-29

**Authors:** Roghayeh Pourkia, Mahsa Panahi, Zahra Emkanjoo, Mozhgan Parsaee, Maryam Shojaeifard, Babak Sattartabar, Yousef Rezaei, Niloufar Samiei

**Affiliations:** ^1^Echocardiography Research center, Rajaie Cardiovascular Medical and Research Center, Iran University of Medical Sciences, Tehran, Iran; ^2^Cardiac Electrophysiology Research Center, Electrophysiology Research Center, Rajaie Cardiovascular Medical and Research Center, Iran University of Medical Sciences, Tehran, Iran; ^3^Heart Valve Disease Research Center, Rajaie Cardiovascular Medical and Research Center, Iran University of Medical Sciences, Tehran, Iran; ^4^Tehran University of Medical Sciences, Tehran, Iran

**Keywords:** Left Atrial Appendage, Transthoracic Echocardiography, Transesophageal Echocardiography, 3-Dimensional Echocardiography, Atrial Fibrillation

## Abstract

***Introduction:*** Cardioembolic events are accompanied by left atrial appendage (LAA) in patients suffering from atrial fibrillation (AF); therefore, the LAA closure is implemented as a preventive strategy. The detection of LAA morphologies and function is a paramount step before establishing the LAA closure. Herein, we sought to determine the morphologic features of the LAA in an Iranian population using echocardiographic evaluation.

***Methods:*** Seventy-two near-normal heart patients were investigated by conducting a cross-sectional study. All patients were examined using the 2-dimensional and 3-dimensional transesophageal echocardiography (2D- and 3D-TEE) method. The anatomical features and functions of LAA were examined. All images were stored and analyzed offline.

***Results:*** The patients’ mean age was 39 ± 15.5 year and 33 (45.8%) were female. The most frequent shape of LAA was wind sock . More LAA lobes was observed in patients with AF compared to those with NSR. In comparison with AF group, the NSR had higher LAA flow velocity (*P * < 0.01). The paroxysmal AF had greater LAA flow velocity and LAA ejection fraction in comparison with the chronic AF (39 ± 19 vs. 75 ± 22, *P * < 0.01; and 49±4 vs. 72±14, *P * < 0.003; respectively). The paroxysmal AF had smaller systolic LAA orifice area in comparison with the chronic AF (*P* < 0.02).

***Conclusion:*** The morphologic features of LAA in Iranian population were within the range of other studies and LAA length and orifice diameters in 2D- and 3D-TEE were consistent. In addition, AF influenced the morphologies and functions of LAA compared to sinus rhythm.

## Introduction

Left atrial appendage (LAA) is the remnant of embryonic atrium,^1^ and ninety percent of clots have been observed in the LAA in patients with non-valvular atrial fibrillation (AF), particularly in cases who has an LAA aneurysmal anomaly.^2^ Therefore, we need to evaluate the LAA in AF cases to risk stratify patients regarding the development of cardioembolic events.^3^

Researchers argue that the LAA orifice can be obstructed through nominally invasive epicardial or catheter-based transseptal techniques to decrease the risk of stroke.^2,3^ Thus, it is necessary to provide precise evidences on the LAA anatomy and dimension before advising the LAA closure devices. One must be aware of the complexity and the variability of shape, size, and number of lobes to avoid misinterpretation and the exact evaluation of LAA closure device.^4-7^ Given the temporary experiences with the implementation of LAA closure devices in Iran, we sought to determine the morphologic features of the LAA in Iranian population using echocardiographic evaluation, 2-dimensional transthoracic and transesophageal echocardiography (2D-TTE and 2D-TEE) as well as 3-dimensional TEE (3D-TEE) techniques, to compare it with the morphologies of other populations and provide an invaluable tool for proper closure device implementation.

## Materials and Methods

### Study protocol and population

In a cross-sectional investigation, 80 consecutive patients who underwent echocardiographic examination were prospectively enrolled. Indications for the echocardiographic evaluation included the assessment of interatrial septum, individuals scheduled to undergo ablation for paroxysmal AF while on sinus rhythm, ruling out endocarditis, and ruling out intracardiac clot in patients with AF. Exclusion criteria were significant diastolic dysfunction, left ventricular ejection fraction >50%, any degrees of mitral valve stenosis, greater than mild degrees of mitral valve regurgitation, and atrial septal defect. The study was conducted in Rajaie Cardiovascular and Medical Research Center, since April 2016 to February 2017. After signing a written informed consent, each patient took part in the study.

### Echocardiographic evaluation

An EPIQ 7 ultrasound scanner (Philips Ultrasound, WA, USA) was used to conduct 2D-TEE and 3D-TEE. The standard 2D transthoracic echocardiographic examination was performed using the Affiniti 70 (Philips Ultrasound, WA, USA). Using 2D images taken from the left ventricular long-axis view, the left ventricular end-diastolic and end-systolic as well as left atrial (LA) diameters were determined. The pulsed wave Doppler in a longitudinal section of the LAA viewed from the transvers scan (70° views) was implemented to determine the LAA empty flow velocity. The average of three successive cardiac cycles in patients having normal sinus rhythm (NSR) and five successive cardiac cycles in patients having AF were used to determine the LAA emptying flow velocity. The 3D images of the LAA in a full-volume HVR mode test in NSR and 3D zoom mode in AF patients were specified from 70° 2D views in a manner that the target area was modified to cover all of the LAA and part of the mitral and aortic valves. Each image had a frame rate of 20-30 frames/sec in a full-volume mode along with a 10 frames/sec in a zoom mode. All images were stored and analyzed offline using Q lab software.

The LAA orifice long diameters and the depth of orifice to a lobe tip in 2D multiple views were specified through the transverse scan (0°, 45°, 90°, and 135° views) at LAA end-diastole, which are the standard assessment of LAA before device implementation for LAA closure procedure. Then, we chose the longest one as a final image. We determined the number of LAA lobes and LAA picture from 135° 2D views.

**Figure 1:**
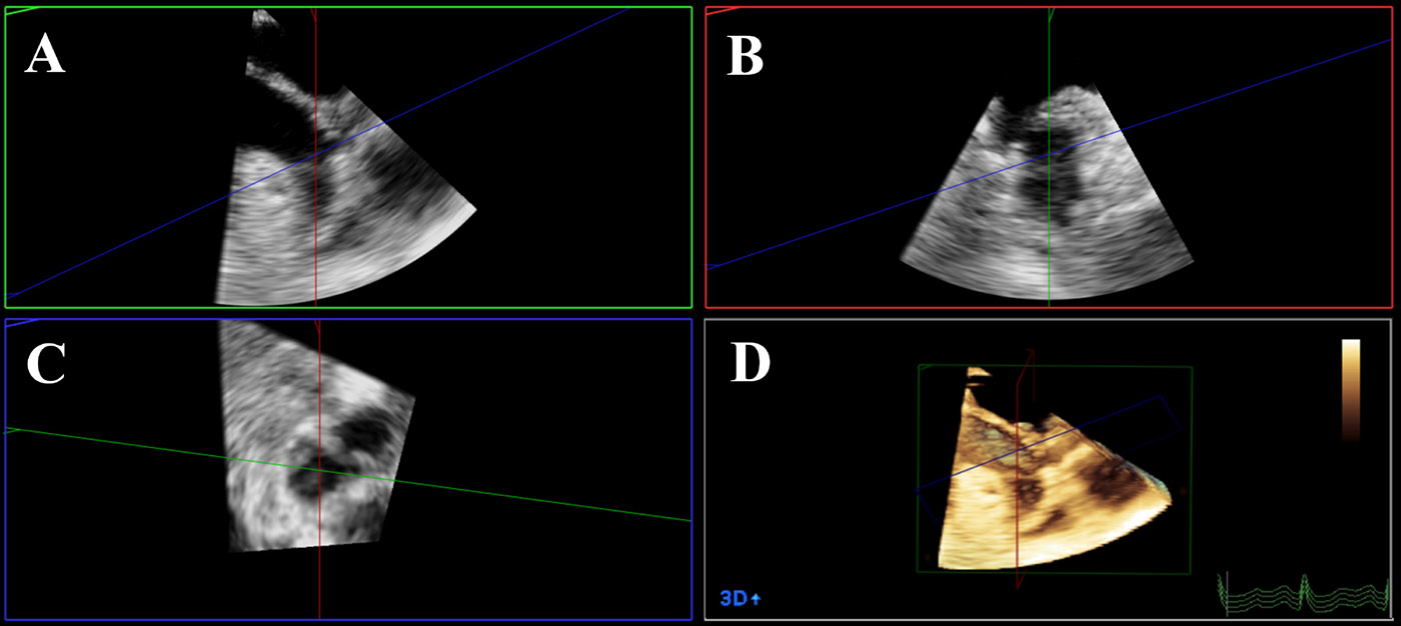
Two- and three-dimensional echocardiographic images of LAA in (A) X and Y axes view, (B) Y and Z axes view, (C) orifice of LAA by MPR, and (D) three-dimensional.

In the 3D imaging analysis where the x-axis plane and the perpendicular y-axis plane both went through the longitudinal section of the LAA, the image was rotated to display the two planes to view the maximum area of LAA in one of these two planes. The LAA orifice long and short diameters and orifice area at LAA end-diastole and end-systole were determined by the implementation of multiplanar reconstruction technique. Using computer software and tracing the endocardium of LAA within the various planes at LAA end-diastole and end-systole, the LAA volume was also measured. Finally, the ejection fraction of LAA was measured using the systolic and diastolic volumes of LAA. We defined the end-systole of LAA as the start of QRS wave and the end-diastole of LAA as the end of T wave on the electrocardiogram (Figure 1).

### Statistical analysis

The distribution of variables was evaluated, and continuous variables reported as mean ± standard deviation or median (interquartile range), as appropriate. Then, those were compared among the groups by conducting the independent *t* test or the Mann-Whitney U test. Frequency was used to represent all categorical variables and they were compared among groups by conducting the χ^2^ test or the Fisher exact test. Two-sided *P* value was calculated for all comparisons.

## Results

**Figure 2:**
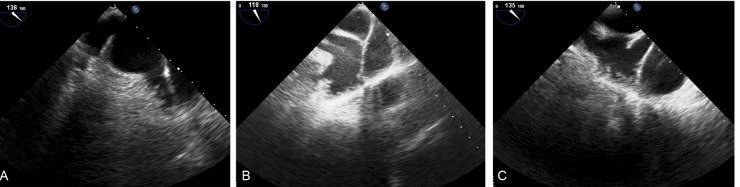
Three morphologies of LAA, including (A) wind sock, (B) broccoli, and (C) chicken wing.

Of 76 patients who underwent echocardiographic examination, four patients were excluded due to poor quality of 3D images (n=4). Table 1 shows baseline characteristics and 2D-TTE measurements. Wind sock (75%), Broccoli (16.7%), and Chicken wing (8.3%) were the shapes of LAA by order in this cohort (Figure 2). Morphologically, the LAA had 1 to 4 lobes; 18 patients had one lobe (25%); 23 patients (31.9%) had two lobes; 24 patients (34.7%) had three lobes; and six patients (8.3%) had four lobes.

**Table 1:** Clinical characteristics and transthoracic echocardiography measurements

	**Total (n=72)**	**NSR (n=50)**	**Paroxysmal AF (n=17)**	**Chronic AF (n=5)**
Age (y)	39 ± 15.5	38 ± 16	52 ± 15	53 ± 12
Sex (F/M)	33/39	25/25	5/12	3/2
BSA (cm^2^)	1.84 ± 0.5	1.8 ± 0.6	1.9 ± 0.7	1.98 ± 0.5
Heart rate	83 ± 14	82 ± 19	78 ± 12	100 ± 8
**2D-TTE parameters**				
Left ventricle end-diastolic diameter (cm)	4.6 ± 0.7	4.5 ± 0.9	5 ± 0.8	4.7 ± 0.8
Left ventricle end-systolic diameter (cm)	3.2 ± 0.4	3.1 ± 0.5	3.4 ± 0.3	3.47 ± 0.8
Left ventricle ejection fraction (%)	52 ± 3	51 ± 4	54 ± 2	49 ± 4
Left atrial diameter (cm)	3.45 ± 0.5	3.3 ± 0.6	3.5 ± 0.4	4.3 ± 0.6

All data are presented as number (%) or mean ± SD AF, atrial fibrillation; BSA, body surface area; TTE, trans thoracic echocardiography; LAA, left atrial appendage.

Table 2 shows comparisons between patients with NSR and those having any AF rhythm (5 patients with AF and 17 patients with paroxysmal AF) with regard to the LAA 2D and 3D measurements. When compared the number of LAA lobes in groups with or without AF rhythm, more lobes were observed in patients with AF rhythm compared to patients with NSR (*P* = 0.05). When compared LAA lobes between both genders, the number of lobes was comparable between genders (*P* = 0.139). In addition, the LAA lobe was categorized into two groups (1 or 2 lobes versus 3 or 4 lobes). The number of individuals with 3 or 4 lobes was significantly higher among males compared to females (53.8% versus 30.3%, respectively; *P* = 0.044).

**Table 2:** Comparisons of left atrial appendage characteristics between patients in normal sinus rhythm and those with all patients with atrial fibrillation

**Variable**	**Total (n=72)**	**NSR (n=50)**	**All AF (n=22)**	***P*** ** value**
**2D-TEE parameter**				
Long LAA diameter	20.6±7	20.7 ± 2.8	20.6 ± 3.4	0.9
Long LAA length	28.3±5	27.6 ± 5	29.9 ± 5	0.08
LAA lobes				0.05
1 lobe	18 (25%)	17 (34%)	1 (4.5%)	
2 lobes	23 (31.9%)	14 (28%)	9 (40.9%)	
3 lobes	25 (34.7%)	16 (32%)	9 (40.9%)	
4 lobes	6 (8.3%)	3 (6%)	3 (23.6%)	
**3D-TEE Parameter**				
Diastolic LAA orifice long diameter (mm)	20.2 ± 3	19.98 ± 3	20.8 ± 3.3	0.27
Systolic LAA orifice long diameter (mm)	13.9 ± 3.6	13.6 ± 3.7	14.5 ± 3.5	0.3
Diastolic LAA orifice short diameter (mm)	13.8 ± 3	13.3 ± 2.9	14.9 ± 3	0.49
Systolic LAA orifice short diameter (mm)	9.2 ± 2.8	8.7 ± 2.9	10.1 ± 2.5	0.5
Systolic LAA orifice area (cm^2^)	1 ± 0.6	0.97 ± 0.6	1.2 ± 0.6	0.09
Diastolic LAA orifice area (cm^2^)	2.2 ± 0.8	2.19 ± 0.78	2.49 ± 0.8	0.15
LAA depth (mm)	30.7 ± 7	29.7 ± 7	33 ± 6.4	0.05
End diastolic volume (mL)	3.4 ± 1.8	3.1 ± 1.5	3.9 ± 2.4	0.09
End systolic volume (mL)	1.2 ± 1.5	1.2 ± 1.8	1.1 ± 0.7	0.9
LAA EF (%)	70.7 ± 14	71.8 ± 14	68.2 ± 12	0.3
LAA flow velocity (cm/s)	70.6 ± 23	75 ± 22	60 ± 22	0.01

All data are presented as number (%) or mean ± SD. NSR, normal sinus rhythm; AF, atrial fibrillation; TEE, Trans esophageal echocardiography; LAA, left atrial appendage; EF, ejection fraction.

The AF group had lower LAA flow velocity than the NSR patients (75 ± 22 cm/s versus 60 ± 22 cm/s *P* < 0.01). When comparing chronic AF and paroxysmal AF, the LAA flow velocity and the LAA ejection fraction were lower in chronic AF compared with paroxysmal AF (39 ± 19 versus 75 ± 22, *P* < 0.01; and 49 ± 4 versus 72 ± 14, *P* < 0.003; respectively). In addition, there was a trend toward AF patients to have more LAA lobes compared to paroxysmal AF (*P* = 0.07). Besides, the paroxysmal AF group had smaller systolic LAA orifice area in comparison with the chronic AF group (1.7 ± 0.9 vs. 0.97 ± 0.8, *P* < 0.02). Other data are summarized in Table 3.

**Table 3:** Comparisons of left atrial appendage characteristics between patients with paroxysmal and chronic atrial fibrillation

**Variable**	**Paroxysmal AF (n=17)**	**Chronic AF (n=5)**	***P*** ** value**
**2D-TEE Parameter**			
Long LAA diameter	20.6 ± 3	20.5 ± 2.5	0.9
Long LAA length	30.2 ± 6	29 ± 3.4	0.7
LAA lobes			0.07
1 lobe	1 (5.9%)	0 (0)	
2 lobes	6 (35.3%)	3 (60%)	
3 lobes	9 (52.9%)	0 (0)	
4 lobes	1 (5.9%)	2 (40%)	
**3D-TEE Parameter**			
Diastolic LAA orifice long diameter (mm)	20.8 ± 3	21 ± 4	0.88
Systolic LAA orifice long diameter (mm)	14 ± 2	16.4 ± 5	0.2
Diastolic LAA orifice short diameter (mm)	14.7 ± 2.9	15.7 ± 3	0.5
Systolic LAA orifice short diameter (mm)	9.6 ± 2	11.9 ± 3	0.09
Systolic LAA orifice area (cm2)	1 ± 0.4	1.7 ± 0.9	0.02
Diastolic LAA orifice area (cm2)	2.4 ± 0.8	2.8 ± 0.7	0.3
LAA depth (mm)	33.2 ± 7	32.7 ± 3	0.9
End diastolic volume (ml)	4.2 ± 2	3.2 ± 1	0.46
End systolic volume (ml)	1 ± 0.6	1.5 ± 0.8	0.25
LAA EF (%)	72 ± 9	49 ± 4	0.003
LAA flow velocity (cm/sec)	66.7 ± 19	39 ± 19	0.01

All data are presented as number (%) or mean ± SD AF, atrial fibrillation; TEE, Trans esophageal echocardiography; LAA, left atrial appendage; EF, ejection fraction

All echocardiographic measurements by 2D-TTE, 2D-TEE, and 3D-TEE in both the NSR and the paroxysmal AF groups were summarized in Table 4. Both groups were not different in terms of the LAA features. Therefore, we considered these two groups as a whole, which is named normal group. The means of LAA characteristics measured by 2D-TEE and 3D-TEE in normal groups are summarized in Table 5. In addition, the ranges of similar values reported in previous studies are summarized.

**Table 4:** Comparisons of left atrial appendage characteristics between patients with normal sinus rhythm and those with paroxysmal atrial fibrillation

**Variable**	**Paroxysmal AF (n=17)**	**NSR (n=50)**	***P*** ** value**
**2D-TEE parameter**			
Long LAA diameter	20.6 ± 3	20.7±2.8	0.9
Long LAA length	30.2 ± 6	27.6 ± 5	0.09
LAA lobes			0.14
1 lobe	1 (5.9%)	17 (34%)	
2 lobes	6 (35.3%)	14 (28%)	
3 lobes	9 (52.9%)	16 (32%)	
4 lobes	1 (5.9%)	3 (6%)	
**3D-TEE Parameter**			
Diastolic LAA orifice long diameter (mm)	20.8 ± 3	19.98 ± 3	0.3
Systolic LAA orifice long diameter (mm)	14 ± 2	13.6 ± 3.7	0.7
Diastolic LAA orifice short diameter (mm)	14.7 ± 2.9	13.3 ± 2.9	0.1
Systolic LAA orifice short diameter (mm)	9.6 ± 2	8.7 ± 2.9	0.2
Systolic LAA orifice area (cm2)	1 ± 0.4	0.97 ± 0.6	0.5
Diastolic LAA orifice area (cm2)	2.4 ± 0.8	2.19 ± 0.78	0.4
LAA depth (mm)	33.2 ± 7	29.7 ± 7	0.08
End diastolic volume (ml)	4.2 ± 2	3.1 ± 1.5	0.05
End systolic volume (ml)	1 ± 0.6	1.2 ± 1.8	0.8
LAA EF (%)	72 ± 9	71.8 ± 14	0.9
LAA flow velocity (cm/sec)	66.7 ± 19	75 ± 22	0.15

All data are presented as number (%) or mean ± SD. NSR, normal sinus rhythm; AF, atrial fibrillation; TEE, Trans esophageal echocardiography; LAA, left atrial appendage; EF, ejection fraction.

**Table 5:** Left atrial appendage characteristics in normal group, including both normal sinus rhythm and paroxysmal atrial fibrillation

**Variable**	**Present study** ^a^	**Prior Studies** ^ b,8-14^
**2D-TEE Parameter**		
Long LAA diameter	20.68 ± 3	19-28
Long LAA length	28.3 ± 5.45	25-37
**3D-TEE Parameter**		
Diastolic LAA orifice long diameter (mm)	20.2 ± 3	19-28
Systolic LAA orifice long diameter (mm)	13.7 ± 3.4	13-21
Diastolic LAA orifice short diameter (mm)	13.7 ± 3	NA
Systolic LAA orifice short diameter (mm)	9 ± 2.7	NA
Systolic LAA orifice area (cm^2^)	1 ± 0.54	1.25-3
Diastolic LAA orifice area (cm^2^)	2.2 ± 0.8	2-4
LAA depth (mm)	30.6 ± 7	25-37
End diastolic volume (mL)	3.4 ± 1.9	3.6-8
End systolic volume (mL)	1.1 ± 1.6	0.9-2
LAA EF (%)	71.9 ± 13	73
LAA flow velocity (cm/s)	73 ± 21	65

^a^All data are presented as mean ± SD ^b^All values are as a range of prior studies (Minimum and Maximum) NSR, normal sinus rhythm; AF, atrial fibrillation; TEE, Trans esophageal echocardiography; LAA, left atrial appendage; NA, not available; EF, ejection fraction.

## Discussion

In this study we showed that the morphological and functional features of LAA in an Iranian population, as the representative of Middle Eastern population, were in lines with the same values reported in other ethnicities. Moreover, our findings underscore how AF rhythm is associated with the morphological features of the LAA. These findings notify us that we should consider this phenomenon in our daily practice, since it can considerably influence our approach to LAA in patients with AF rhythm.

In this study the 2D-TEE was conducted to investigate the LAA orifice size along with 3D echocardiography; it should be emphasized that due to its natural character, the 2D-TEE is a limited technique. Conducting the 2D-TEE requires the multiple views of the measurement. Moreover, it is likely that foreshortening and limited echo planes bring about inaccurate estimation of the LAA orifice size, as the results of present study showed that there were not 0 views in 12.5% of the patients. Thus, other 3D imaging procedures like computed tomography (CT), cardiac magnetic resonance, and real-time 3D-TEE have been proposed.^8,9^ However, there is a correlation between CT and the non-negligible radiation exposure. Moreover, cardiac magnetic resonance is not cheap and cannot be found easily. It is worth noting that these two techniques cannot be conducted at bedside and they are not capable of yielding real-time images of the LAA. Thus, it is likely that real-time 3D-TEE could modify the first-line approach because it is capable of yielding the real-time 3D views of the LAA without radiation exposure and contrast administration.^8^ 3D-TEE was able to show the LAA visually in most of the patients in the present study. Likewise, the desirable 3D images of LAA were not yielded in 5.5% of patients in the first report on the LAA visualization by 3D-TEE.^10^ Further large-scaled studies in different populations may provide valuable data regarding the morphologies of LAA by 3D imaging modalities and the subsequent use in the LAA closure by devices.

In our setting, since the full-volume HVR modality was not available for AF patients, zoom-mode imaging was implemented. The thorough morphological variations of LAA in an Iranian population have been shown in present study. The means of LAA orifice diameter in long axis and LAA length were 20.6 ± 3 mm and 28.3 ± 5 mm, respectively. All these measures were in the ranges of prior studies in other populations (LAA long diameter = 19-28 mm and LAA length = 25-37 mm).^8-14^ Given these findings, it seems that the ranges of basic morphologic parameters to determine LAA device closure size are identical in different population. However, it was supposed that all existing occluders were round, but it is likely that a round implant over an oval-shaped orifice (typically observed in this subgroup of AF patients) causes flawed closure of the orifice leading to residual leaks.^10,16^ These findings suggests that during the development of AF, some structural changes can occur in the LAA, and the evaluation of such a phenomenon is proposed in an Iranian population in current report.

This study revealed that the LAA lobes are changeable. To determine the number of LAA lobes, it is necessary to distinguish them from the pectinate muscle, since assessments may be difficult in patients with multi-lobe LAA and concomitant large pectinate muscle. In this setting, 3D-TEE can be of utmost importance to evaluate complex morphologies.^15^ The clinical implication of LAA lobe variability is unknown. There was a correlation between the complex LAA morphology specified by an added number of LAA lobes (≥3) and the existence of LAA thrombus with no dependency on clinical risk and blood stasis.^16^ In addition, Wang et al^17^ found that the number of LAA lobes were associated with the LA thrombus and LA spontaneous echo contrast during echocardiography. In the current study we found that number of lobes was higher in AF patients, and there was even a trend toward AF patients to have more lobes compared to paroxysmal AF. We think that the increased chance of LA thrombus and subsequent stroke may be mediated, in some way, by the number of LAA lobes in patients with AF. On the other hand, someone might postulate that the morphology of LAA lobes is the underlying pathogenesis of AF development. Further prospective studies in normal populations can provide us invaluable information in the setting of such phenomenon.

It is likely that 3D-TEE helps recognize the implications of LAA lobe variability.^13^ Moreover, various shapes of the LAA were investigated in the present study; the results show that these shapes were somewhat similar to other studies. In our study, 2D-TEE was used to determine the LAA dimension; then, they were compared with the results of 3D-TEE. To specify the size of the LAA device, two major criteria namely the LAA orifice long diameter and depth were applied; the results show that they were not different significantly. Prior studies using TEE have revealed that LAA diameter, length, and surface area were greater in patients with persistent AF than in individuals with sinus rhythm. The LAA contractile dysfunction evaluated by TEE has also been reported. The LAA functional abnormalities in chronic AF were associated with an increased risk for the formation of clots within the LAA. In addition, the LAA dysfunction can anticipate the recurrence of AF in patients undergoing cardioversion.^3,12,18,19^ Akdeniz et al^20^ stated that LAA flow velocity provides useful information that help predict the cardioversion result in AF patients before TEE-guided cardioversion. In addition, Aslanabadi et al^1^ observed that percutaneous transvenous mitral commissurotomy boosts the LAA function as it improved the LAA ejection fraction and emptying velocity after intervention in patients with rheumatic heart disease independent of rhythm status (NSR versus AF). In contrast, Alizadeh et al^21^ showed that the implementation of pacing using VVI mode impaired the LAA function, but it did not increased the thrombus formation within the LAA. These findings designate that LAA remodeling in individuals with AF happened during the transition from sinus rhythm to fibrillation. In the present study, the LAA ejection fraction and flow velocity decreased and systolic LAA orifice area increased in the AF group compared with the normal group. These findings are consistent with previous studies and underscore the need for further studies to explore the function and morphology of LAA.

### Study limitations

This study suffers from some limitations. First, our group included a small number of patients. Second, the echocardiographic findings were not validated against other diagnostic imaging modalities such as cardiac multi-detector CT or magnetic resonance imaging. Third, due to lack of follow-up, we did not provide information regarding the effects of morphologic features of LAA on patients’ prognosis, particularly in those with AF. Finally, we did not record the duration of AF in our cohort, yet all patients were near normal heart, which justifies this difference.

## Conclusion

This study revealed that the sizes of LAA orifice and length diameters in Iranian population, as the representative of Middle Eastern population, were within the ranges of studies from other ethnicities. The measurements of LAA length and orifice diameter with 2D- and 3D-TEE were consistent; however, there were some morphologic and functional changes in LAA parameters in individuals with AF compared to patients in sinus rhythm.

## Competing interests

None.

## Ethical approval

The ethics committee of Rajaie Cardiovascular Medical and Research Center, Tehran, Iran, approved the study protocol (Ethical code number: IR.IUMS.FMD.REC.1396.9311171008).
